# Targeting Pyrimidine Metabolism in the Era of Precision Cancer Medicine

**DOI:** 10.3389/fonc.2021.684961

**Published:** 2021-05-28

**Authors:** Wanyan Wang, Jiayan Cui, Hui Ma, Weiqiang Lu, Jin Huang

**Affiliations:** ^1^ State Key Laboratory of Bioreactor Engineering, Shanghai Key Laboratory of New Drug Design, School of Pharmacy, East China University of Science and Technology, Shanghai, China; ^2^ Shanghai Key Laboratory of Regulatory Biology, Institute of Biomedical Sciences and School of Life Sciences, East China Normal University, Shanghai, China

**Keywords:** metabolic reprogram, pyrimidine metabolism, precision medicine, dihydroorotate dehydrogenase, pyrimidine inhibitor

## Abstract

Metabolic rewiring is considered as a primary feature of cancer. Malignant cells reprogram metabolism pathway in response to various intrinsic and extrinsic drawback to fuel cell survival and growth. Among the complex metabolic pathways, pyrimidine biosynthesis is conserved in all living organism and is necessary to maintain cellular fundamental function (i.e. DNA and RNA biosynthesis). A wealth of evidence has demonstrated that dysfunction of pyrimidine metabolism is closely related to cancer progression and numerous drugs targeting pyrimidine metabolism have been approved for multiple types of cancer. However, the non-negligible side effects and limited efficacy warrants a better strategy for negating pyrimidine metabolism in cancer. In recent years, increased studies have evidenced the interplay of oncogenic signaling and pyrimidine synthesis in tumorigenesis. Here, we review the recent conceptual advances on pyrimidine metabolism, especially dihydroorotate dehydrogenase (DHODH), in the framework of precision oncology medicine and prospect how this would guide the development of new drug precisely targeting the pyrimidine metabolism in cancer.

## Introduction of Pyrimidine Metabolism Pathway

In mammal, pyrimidine can be produced through *de novo* synthesis pathway taking amino acids as substrates or salvage pathway by uptake of the circulating pyrimidines in the bloodstream. Generally, salvage pathway is the main pyrimidines sources for resting or fully differentiated cells, while the *de novo* pathway is necessary for high-proliferating cells to meet the boosted requirement of pyrimidines. Uridine 5′-monophosphate (UMP) is the first production of *de novo* pyrimidine pathway and is further converted to other pyrimidine nucleosides for synthesis of DNA and RNA. On the other hand, pyrimidine synthesis is also implicated in other metabolic pathways. Cytidine triphosphate (CTP) acts as a shuttle for PtdCho through cytidine diphosphate (CDP)-choline pathway (1). Uridine-5′-triphosphate (UTP) participates in the formation of UDP-N-acetylglucosamine (UDP-GlcNAc), UDP-glucose, UDP-galactose, and UDP-glucuronic acid (2). Owing to the critical functions of pyrimidine in cell proliferation and survival, the disorder of pyrimidine metabolism has been considered as a vital driver in tumor initiation and progression (3, 4).

### Pyrimidine Biosynthesis Through *De Novo* Pathways

**Figure 1:**
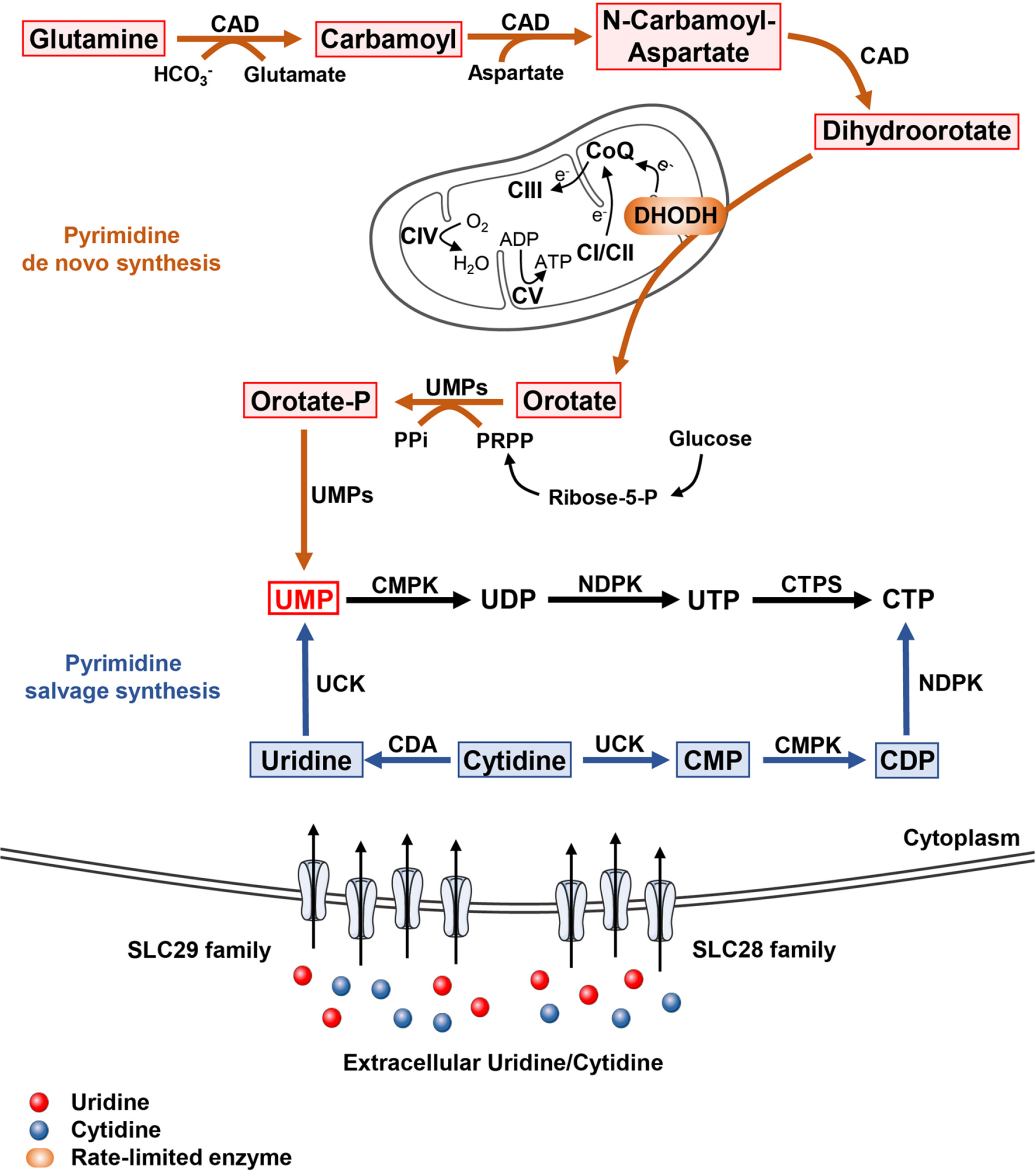
The pyrimidine metabolism in cells In mammal, pyrimidine synthesis is composed of *de novo* pathway and salvage pathway. The *de novo* synthesis pathway initiated with the generation from glutamine to dihydroorotate catalyzed by trifunctional enzyme CAD including carbamoyl-phosphate synthetase, aspartate transcarbamylase, and dihydroorotase. The mitochondrial inner membrane protein DHODH oxidizes dihydroorotase to orotate accompanied by the respiratory chain transmission. Orotate is subsequently phosphorylated and produced to UMP by bifunctional enzyme UMPs. As for pyrimidine salvage synthesis pathway, cells obtained extracellular uridine and cytidine mainly through two nucleosides transporter families, SLC28A as well as SLC29A. Subsequently, uridine and cytidine are converted to UMP and CTP, respectively.

Pyrimidine *de novo* biosynthesis pathway is initiated by a trifunctional enzyme CAD (carbamoyl phosphate synthetase (CPS), aspartate carbamoyltransferase (ATC) and dihydroorotase) (**Figure 1**) **(** 5). The CPS leads the first reaction using glutamine, bicarbonate, and ATP to produce carbamoyl phosphate, which is the first committed step in pyrimidine *de novo* synthesis metabolic flux (6). The second step is catalyzed by the ATC domain *via* converting aspartate and carbamoyl phosphate to carbamoyl aspartate (7). The dihydroorotase domain, a Zn metalloenzyme locating between CPS and ATC domains, hydrolyzes carbamoyl aspartate to dihydroorotate (DHO) (8). CAD, governing the initiation of pyrimidine *de novo* synthesis pathway (9–11), is under precise control in cells (12, 13).

Dihydroorotate dehydrogenase (DHODH), a ubiquitous flavoprotein (flavin mononucleotide, FMN) located in the mitochondrial inner membrane, converses DHO to orotic acid (14). DHODH is a unique enzyme that could perform this conversion in cells, representing the second rate-limiting step in pyrimidine *de novo* synthesis pathway. Additionally, DHODH transfers two electrons to coenzyme Q (CoQ) *via* ubiquinone and directly couples to the mitochondrial respiratory chain and oxygen consumption (15–18).

Subsequently, uridine monophosphate synthetase (UMPS), a bifunctional protein, transforms orotic acid into uridine monophosphate (UMP) through two catalytic reactions. The N-terminal domain of UMPS converts orotic acid into orotidylate (OMP) taking phospho-α-dribosyl-1-pyrophosphate (PRPP) as co-substrate, while C-terminal converts OMP into UMP (19).

In general, the *de novo* pyrimidine flux is low in resting or fully matured cells, where the demand of pyrimidines is mostly satisfied through the salvage pathway. Nevertheless, the high pyrimidine flux is indispensable in cancer cells in order to meet their increased need of nucleic acid and other cellular components (4, 20). Besides, owing to the bifunction of DHODH linking the pyrimidine *de novo* synthesis pathway with mitochondrial respiratory chain and oxygen consumption, this pathway becomes a pacemaker for cell growth and proliferation under limited oxygen tension. Consequently, pyrimidine *de novo* synthesis represents a promising therapeutic target in delaying cancer progression (15, 21–25).

### Pyrimidine Biosynthesis Through Salvage Pathways

Although *de novo* pathway generally provides competent pyrimidine for cells growth and development, energetic expensiveness of this pathway restricts its application in cells (26). Cells at rest can meet their pyrimidine requirement *via* salvage pathway using intracellular nucleic acid degradation product or extracellular nucleoside pool in the blood stream (**Figure 1**) (27).

Intracellularly, uridine/cytidine kinase (UCK) participates in the recycling of cytidine and uridine to CMP and UMP, respectively. CMP is then converted to CTP by cytidine monophosphate kinase (CMPK) and nucleoside-diphosphate kinase (NDPK). For reusing extracellular nucleoside, their availabilities between cells and external environment are primarily mediated by specific nucleosides transport channels and pumps (28, 29). Specifically, nucleoside transport in mammals is comprised of two major gene families, SLC28 (SLC28A1, SLC28A2, SLC28A3) for encoding cation dependent concentrative nucleosides transporters (CNTs) and SLC29 (SLC29A1, SLC29A2, SLC29A3, SLC29A4) for encoding energy independent equilibrate nucleoside transporters (ENTs) (28, 29). These nucleosides transporters differ both in concentration of cation coupling and permeant selectivity. Also, targeting pyrimidine salvage pathway was considered as a valuable approach for reducing pyrimidines accessibility in cancer cells (30–36).

### Biological Role of Pyrimidines

As an non-negligible substrate for DNA and RNA biosynthesis, UMP can be phosphorylated to generate produce UDP and UTP (37). UDP is catalyzed into deoxy-UMP (dUMP), which is the substrate to produce deoxyribonucleotides dTMP and dTTP through thymidylate synthase (TS). CTP synthetase (CTPS) converts UTP into CTP using glutamine as amine donor (12). Overall, UMP can supply all kinds of pyrimidine deoxyribonucleotides (dTTP, dCTP) and ribonucleotides (CTP, UTP) for DNA and RNA biosynthesis to sustain transmission of genetic information in cell proliferation (3, 38).

Beyond DNA and RNA biosynthesis, CTP also participates in phospholipids biosynthesis in cells. Phosphatidylcholine (PC) is a primary component of phospholipid among biological membranes as well as a source of lipid-like second messengers (39), indicating that PC is a vital substance in cell metabolism and growth. CDP-choline generation is a rate-limiting step for the PC biosynthesis in mammals, which is produced by choline phosphate cytidylyltransferase (CCT) using CTP and choline phosphate (40). Thus, intracellular CTP pool is critical for the generation of PC and the subsequent phospholipid biosynthesis.

During energy metabolism, glucose-derived glucose-1-phosphate combined with UTP to produce UDP-glucose, which is further catalyzed to UDP-glucuronic acid through UDP-glucose 6-dehydrogenase (UGDH) (41, 42). UDP-glucuronic acid is a requisite precursor for the glycosaminoglycans, a component of proteoglycans for sustaining electrolyte as well as fluid balance in the extracellular matrix.

On the other hand, UTP also takes part in the glycosylation modification of proteins, which has a wide range of function and shows essential role for life even at the single cell level (43). In this case, UTP combines with N-acetylglucosamine (GlcNAc) derived from hexosamine biosynthesis (HBP) pathway involving four enzymes converting fructose 6-phosphate to UDP-GlcNAc, to generate UDP-GlcNAc. UDP-GlcNAc is a necessary substrate for O-linked glycosylation (O-GlcNAcylation), a frequent and essential post-translation modification of protein in linking glycosidic of saccharides to proteins in Golgi apparatus (44). During this process, the O-GlcNAc transferase (OGT) takes UDP-GlcNAc as a substrate to install GlcNAc to the hydroxyl oxygen of serine or threonine residues of proteins (45), while O-GlcNAcase (OGA) catalyzes the removal of O-GlcNAc from modified proteins (46, 47).

Together, these evidences demonstrate that pyrimidine metabolism cooperates with various cell metabolism and signaling pathways, demonstrating the decisive effects of pyrimidine metabolism in determining cell fates including proliferation, differentiation, apoptosis, and metastasis.

## Oncogenes Reprogram Pyrimidine Metabolism in Cancers

### Dysfunction of Pyrimidine Metabolism in Cancers

**Figure 2:**
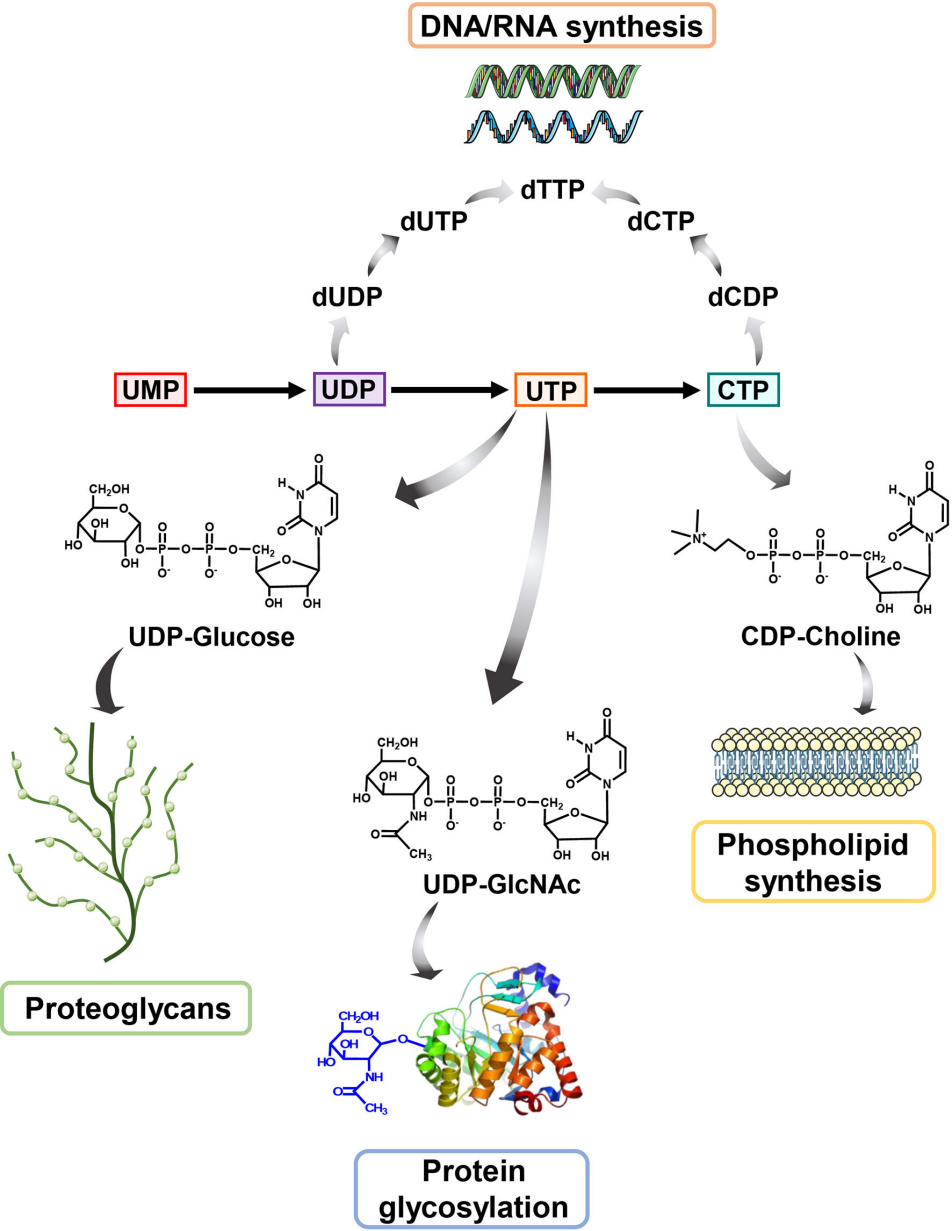
The biological role of pyrimidine In addition to serving as an indispensable substrate for DNA and RNA biosynthesis, pyrimidine also participates in many other cellular metabolisms. UDP-GlcNAc, derived from UTP, is a pivotal metabolic used in O-GlcNAc modification of protein while UDP-glucose is used to synthesis proteoglycans. Besides, CTP could be converted to CDP-Choline for participating in producing phosphatidylcholine (PC), a primary component of phospholipid.

In cancer, dysfunctional pyrimidine *de novo* synthesis not only supplies high-level of nucleotides, but also affects other metabolism pathways and cellular signaling (**Figure 2**). For instance, the essential characters of CTP in phospholipid synthesis, UDP in glucose metabolism and UTP in protein glycosylation hint the influence of pyrimidine synthesis in tumorigenesis. The high requirement of membrane components in active cells sets the indispensable of phosphatidylcholine obtained from CTP and choline in membrane biogenesis. It is reported that phospholipid synthesis pathway was highly relied on the cellular CTP level, which modulates the mitochondrial phospholipid composition upon altered abundance of ether lipids (18). Moreover, CTP also takes part in signaling transmission, enzymatic processes of membrane as well as cell homeostasis (48–51).

Glycosaminoglycan, derived from UDP and glucose, resides in the extracellular aiming to provide mechanical support for cells, involving in the cell migration, adhesion, motility and wound healing (52). The elevated glycosaminoglycan concentration is identified in multiple types of cancers, such as breast cancer, brain tumor, glioblastoma, and lung cancer (53–56). During the glycosaminoglycan biosynthesis, UGDH, catalyzing conversion of UDP-glucose to UDP-glucuronate, is recognized as the rate-limiting enzyme and has been identified as a potential anti-cancer target (55, 57–60). While, the precursor contents of glycosaminoglycan were rarely investigated. Recently, Xiongjun Wang et al. found that the activation of UGDH by epidermal growth factor receptor (EGFR) attenuates the UDP-glucose-mediated disruption of the interaction of HuR and SNAI1 mRNA, which promotes lung metastasis through initiating the epithelial-mesenchymal transmission (54).

UTP is critical for protein glycosylation by serving as a glycosylation substrate sub-fraction. It is reported that protein glycosylation level is generally elevated in various malignancies and has been identified as a hallmark of cancer (61). As an abundant post-translational modification of protein, glycosylation takes part in sustaining cancer cell growth and proliferation (62–69). O-GlcNAc modification is important for VEGF levels, and disordered glycosylation of VEGFR could regulate its interaction with galectins, which are associated with both angiogenesis and tumor metastasis (70, 71). The O-GlcNAc was also reported to be implicated in the regulation of oncogenic gain-of-function of mutant p53 (72, 73). Additionally, Kevin Qian et al. demonstrated that ERK signaling pathway profoundly affected O-GlcNAc homeostasis *via* OGA-mediated OGT transcription in pancreatic ductal adenocarcinoma (PDAC), laying the profound role of O-GlcNAc signaling in cancers (74). Decreased O-GlcNAc levels elevates the cell-cycle regulator p27Kip1 and reduces FoxM1 as well as its downstream target genes expression in breast cancer (67). Moreover, Christina M. Ferrer et al. uncovered that O-GlcNAcylation regulates HIF-1α proteasomal degradation *via* a α-ketoglutarate-dependent manner (75). The pentose phosphate pathway (PPP) is critical for rapidly proliferational cells through maintaining cellular redox homoeostasis and the upregulation of PPP has been appeared in various cancer types (76, 77). The activity of G6PD, the rate-limiting enzyme of PPP, was reported to be regulated by O-linked glycosylation of Ser 84, which significantly elevated PPP metabolism flux in human lung cancer cells related to hypoxia pressure regulation (78). Besides, O-GlcNAcylation inhibits phosphofructokinase 1 (PFK1) activity in response to hypoxia, redirecting glucose flux through PPP to confer growth advantage to lung cancer cells (79).

The chronic inflammatory response plays an essential role in cancer development and abundant evidences shown that glycosylation can modulate multiple key mediators involved in the inflammatory process. It is reported that O-linked glycosylation can upregulate the transcription level of NF-κB (80) and pro-inflammatory factor COX2 (81). Furthermore, the protein glycosylation level of COX2 is associated with the therapeutic efficiency of its inhibitors (82). On the other hand, the pro-inflammatory cytokines in turn balance glycan composition of cells *via* increasing glycosyltransferases expression and promote cancer-related antigens biosynthesis in pancreatic cancer as well as gastric cancer (83, 84).

Published studies have suggested that cellular O-glycosylation level is highly associated with the activities of OGT or OGA and the level of substrate UDP-GlcNAc (85–88). However, the direct and detailed role of pyrimidine synthesis in the regulation of O-glycosylation is not well established. Thus, the specific mechanism of pyrimidine synthesis in regulating O-glycosylation needed to be further explored.

Additionally, pyrimidine synthesis also participates in tumor metastasis. Pharmacological inhibition of DHODH was reported to reduce liver metastasis in colorectal cancer (89) and small cell lung cancer model (88). Siddiqui, A. et al. reported that thymidylate synthase (TS) was reported to be increased in cancer cells with a mesenchymal phenotype (90). Depletion of TS significantly decreased the expression of ZEB1, a powerful epithelial-mesenchymal transition (EMT) driver, and impeded the migration of cancer cells. Moreover, TS maintains the de-differentiated state of triple negative breast cancers (91) and drives the EMT phenotypes in NSCLC (92). These results further demonstrated the potential association of pyrimidine metabolism to the malignant process of cancer.

### Oncogene Reprograms Pyrimidine Metabolism in Cancers

Generally, tumorigenesis is accompanied by enhanced metabolic reprogramming for satisfying cells survival and proliferation in front of their internal and external surrounding microenvironment changes. It is well known that cancer cells intend to remodeling metabolism to increase pyrimidine *de novo* synthesis flux to support vigorous growth. Here, we regularly review the influence of various oncogenic signaling pathways on gene expression and post-translational modifications in pyrimidine metabolism in cancers.

#### Oncogenic Signaling Pathways Modulate Gene Transcription in Pyrimidine Metabolism

**Figure 3:**
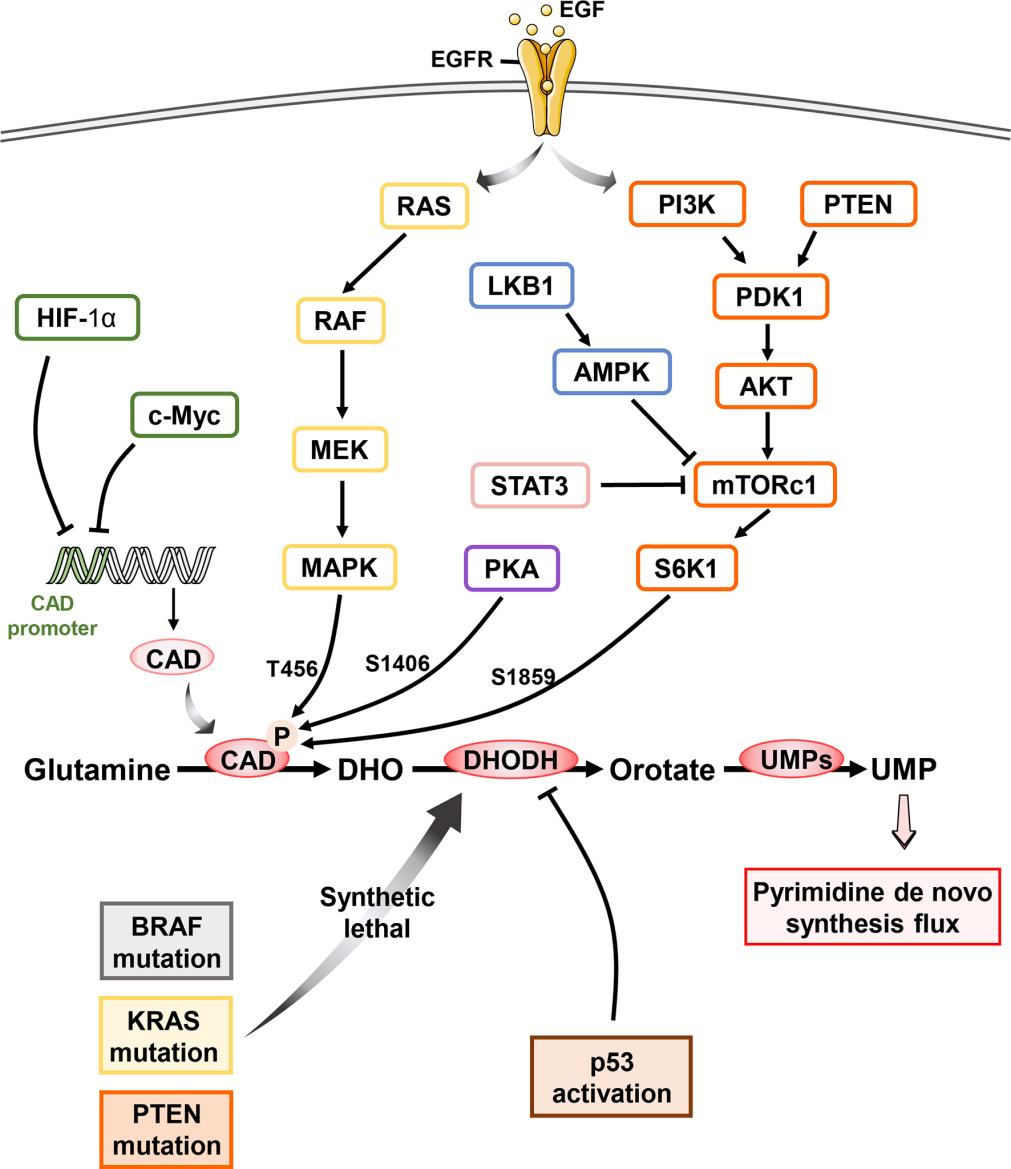
Oncogene reprogram pyrimidine metabolism in cancer Pyrimidine metabolism in cell is regulated by multiple oncogenes and suppressors. Upon the activation of epidermal growth factor, c-Myc dictates the CAD transcription by binding the CAD promoter while HIF-1α exhibits the same pharmacological effect without the participation of EGF. RAS-RAF-MEK-MAPK signaling pathway mediates the phosphorylation of CAD at T456 while PKA phosphorylates S1406 of CAD. Besides, mTOC1 activation following the PI3K/Akt signaling results in the S6K1-regulated phosphorylation of CAD at S1859, which is also inhibited by LKB1 and STAT3 pathways. Both modifications of CAD stimulate the pyrimidine *de novo* pathway flux and promote the pyrimidine synthesis. Cancer cells with BRAF, KRAS as well as PTEN mutation dependent more on pyrimidine *de novo* pathway and exhibit synthetic lethal vulnerability upon DHODH inhibitor. Most importantly, p53 activation is potentially related to the dysfunction of DHODH.

Oncoprotein c-Myc is critical for cell proliferation, differentiation, apoptosis, and cell metabolism *via* regulating the expression of hundreds of target genes (93–95). It was reported that c-Myc upregulated the level of CAD by binding to the highly conserved palindromic E box sequence downstream of transcriptional site of CAD promoter (96–98) (**Figure 3**). Besides, c-Myc is also implicated in the transcription regulation of UMPS and CTPS (99). On the other hand, our studies and others have revealed that intervention of pyrimidine synthesis by targeting DHODH resulted in decreased protein stability and transcription activity of c-Myc in AML and melanoma (100, 101). These studies highlight the complex interplay between c-Myc oncogenic signaling and pyrimidine metabolic reprogramming in cancers (24, 99, 102, 103).

Hypoxia-inducible factor (HIF-1α) was identified to bind to CAD promoter and repress its transcription of *in vivo*, triggering the inhibition of pyrimidine *de novo* synthesis and subsequently cell-cycle arrest (104, 105). In addition, specificity protein 1 (Sp-1) interacts with estrogen receptor α (ERα) physically and results in transcriptional activation of estrogen-responsive genes expression including CAD (106–110).

#### Oncogenic Signaling Pathways Affect Post-Translation Modification of Pyrimidine Metabolism Enzyme

Post-translation modification of pyrimidine pathway is also regulated by various oncogenic signaling pathways. As a hexamer of a 243 KD polypeptide, CAD folds into three functional domains autonomously (111), including CPS, aspartate ATC and dihydroorotase. The activity of CPS domains, governing the flux of pyrimidine *de novo* synthesis, can be reversible inhibited by UTP and allosterically activated by PRPP. Lee M. Graves et al. described that EGFR-mitogen-activated protein (MAP) kinase cascade promotes cell proliferation *via* phosphorylation of CAD at Thr456 (**Figure 3**) **(** 112). This allosteric regulation abolishes the feedback inhibition of UTP as well as endow more sensitive to PRPP, which increases the flux of pyrimidine *de novo* synthesis (113). While, protein kinase A (PKA) induced phosphorylation of Ser1406 elevated UTP inhibition and decreased sensitivity upon PRPP, consequently decreased CAD activity and pyrimidine flux (114). Additionally, both MAPK and PKA complexes with CAD stably and the steric interference by the bound kinase will influence each other (115). Thus, the reciprocal phosphorylation of CAD is under the precise control of PKA and MAPK signaling cascades, which provides the elegant mechanism to regulate pyrimidine *de novo* synthesis.

The mammalian target of rapamycin complex 1 (mTORC1) is a Ser-Thr kinase sensing growth signals to modulate cell metabolism and proliferation (116). PI3K-PTEN-mTORC1 signaling pathway has been shown to phosphorylate Ser1859 of CAD *via* downstream ribosomal protein S6 kinase 1 (S6K1) and promoted CAD oligomerization, facilitating the concerted action of three enzymatic domains (6, 111) and stimulating pyrimidine *de novo* synthesis (10, 117). Karina N. et al. found that loss of sirtuin 3 (SIRT3), a tumor suppressor, also hyperactivates mTORC1-CAD axis and hence increases *de novo* pyrimidine synthesis (118), however the specific mechanism is still to be explored. Furthermore, LKB1 suppressed CPS1 transcriptional level through AMPK, which is known to inhibit mTOR and KRAS. LKB1 mutation speeds the dependence on pyrimidine synthesis in lung cancer cells (13). In addition, loss of argininosuccinate synthase (ASS1) increases CAD activity *via* mTOR-S6K1 signaling pathway and facilitates pyrimidine synthesis to support proliferation (7).

### Targeting Pyrimidine Metabolism Vulnerability in Oncogene-Driven Cancers

A plethora of studies have showed that oncogene-driven cancers exhibit higher level of pyrimidine *de novo* pathway, pointing out the opportunity of precision cancer therapy by targeting the pyrimidine dependence (**Table 1**). In recent years, DHODH has emerged as a promising synthetic lethal target for various oncogenic events, including BRAF (V600E) mutation (101), PTEN deficiency (23) (22), and RAS mutation (119) (**Figure 3**).

**Table 1:** Small molecular inhibitors of pyrimidine metabolic pathway approved by FDA.

Drug	Structure	Target	Disease
Teriflunomide	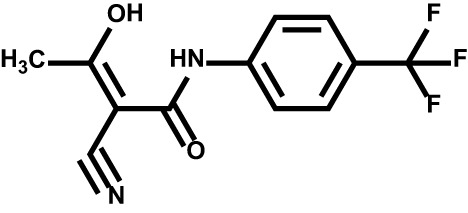	DHODH	Multiple sclerosis
Leflunomide	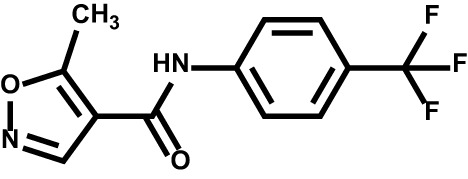	DHODH	Rheumatoid arthritis
Fluorouracil	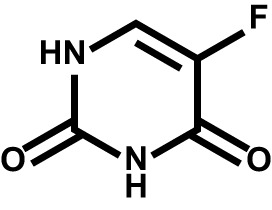	Thymidylate synthase; DNA; RNA	Multiple solid tumors
Floxuridine	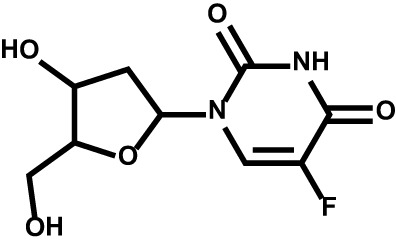	Thymidylate synthase	Metastatic hepatic cancer Stage 4 gastrointestinal adenocarcinoma
Gemcitabine	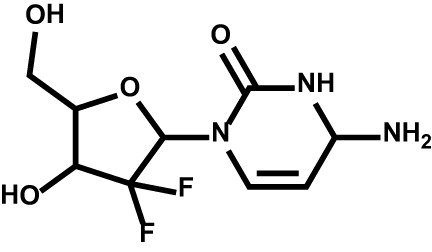	DNA; RRM1; TYMS; CMPK1	Non-small cell lung cancer Pancreatic cancer Bladder cancer Breast cancer

In BRAF (V600E) mutant melanoma cells, DHODH modulates transcriptional elongation and its inhibition completely abrogated cell growth, providing the potential therapeutic strategy through combination of DHODH and BRAF (V600E) inhibitors (101). PTEN, one of the commonly mutated tumor suppressor, is an important negative regulator of oncogenic PI3K signaling (120). It is reported that DHODH inhibition caused inherent defects in DNA repair and accumulated DNA damage contributes to synthetic lethality in PTEN mutant TNBC cells (23). Besides, Kristin K. Brown et al. characterized adaptive metabolic reprogramming of pyrimidine *de novo* synthesis as an early issue promoting chemotherapy resistance in TNBC treatment. Thus, targeting DHODH-driven pyrimidine synthesis seems to be a feasible strategy in overcoming chemotherapy resistance in TNBC clinically (22).

KRAS mutation is one of the most common mutation in human cancer. Previous studies have demonstrated that KRAS mutants induce pyrimidine metabolism reprogramming in multiple malignancies types (121, 122). Malvika Koundinya et al. have reported that KRAS mutant cells seems to exhibit high sensitivity to DHODH inhibitors (119). Decreased expression of mutant KRAS has been shown to downregulate the genes transcriptional level in pyrimidine pathways (123, 124). KRAS mutation activates classic MAPK pathway, which results in MYC upregulation and transcriptional level of the non-oxidative PPP gene ribose 5-phosphate isomerase A (RPIA), promoting the nucleotide biosynthesis (123). Notably, interference with nucleotide biosynthesis resulted in glutamine deprivation in KRAS-driven cancer cells and thus lead to S-phase cell cycle arrest and DNA replication stress (125). These evidences fully define the increase pyrimidine synthesis flux in KRAS mutant cancer cell. On the other hand, although KRAS mutant cells exhibits decreased oxidative phosphorylation (126), the mitochondrial electron transport *via* DHODH in sustaining hyperpolarized mitochondrial membrane potential and pro-survival (127) could explain for the increased sensitivity of KRAS mutant cells to DHODH inhibition.

In addition, p53 also plays a pivotal role in regulating cellular metabolism (128–132). For instance, Irem Kaymak et al. stated that p53 deficiency activates the mevalonate pathway through SREBP2 and speeds ubiquinone synthesis which is crucial to the DHODH catalyzed pyrimidine synthesis (133). Combination of DHODH and CHK1 inhibitors demonstrate synergistically effect in p53 dysfunctional cancer cells through inducing aberrant cell cycle and massive cell death (134). While, the potential association between p53 and DHODH should to be further elaborated.

In addition, cancer cells with electronic transmission chain (ETC) deficiency displayed the dysfunction of DHODH due to the lack of complex III. Restoration of ETC recovers DHODH activity and cell growth (15, 17). These results suggested the indispensable role of DHODH-driven pyrimidine synthesis in linking ETC and tumorigenesis. Furthermore, David B. Sykes et al. described that DHODH inhibitors significantly decreased levels of leukemia-initiating cells, and improved survival of leukemia-bearing mice. This pioneering study defined DHODH as a metabolic regulator of differentiation in multiple subtypes of acute myeloid leukemia (AML) and DHODH inhibition emerged as a potential strategy for overcoming differentiation blockade in treating AML (135). Of note, multiple DHODH inhibitors were found to promote AML cell differentiation and launched to clinical research in AML treatment (**Table 2**) **(** 100, 136–139).

**Table 2:** Representative small-molecular inhibitors of DHODH.

Drug	Structure	Disease	Phase
Brequinar	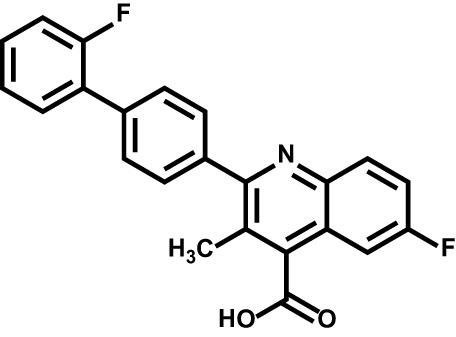	Acute myeloid leukemia	Phase II
BAY2402234	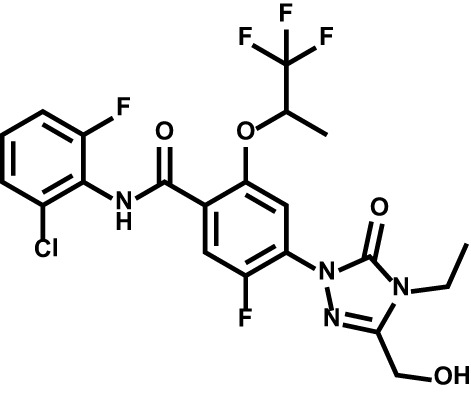	Leukemia	Phase I^a^
ASLAN003	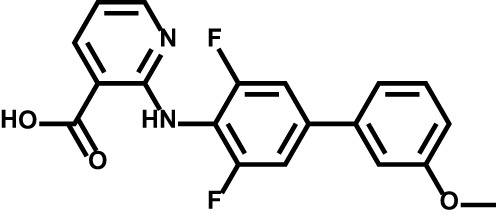	Acute myeloid leukemia	Phase II
PTC299	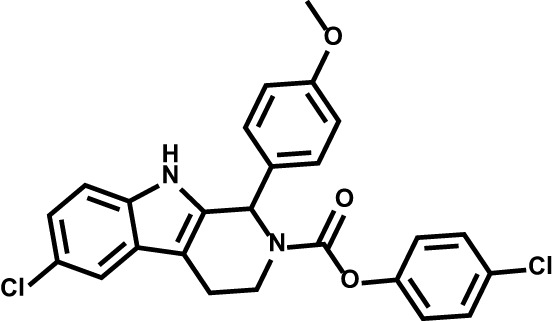	Acute myeloid leukemia	Phase I
AG-636	Unavailable	Lymphoma	Phase I
Leflunomide	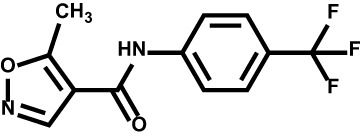	Relapsing multiple sclerosis COVID-19	Phase II
ML390	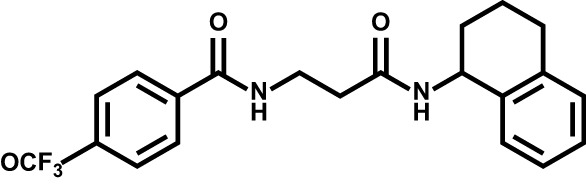	Acute myeloid leukemia	–
Isobavachalcone	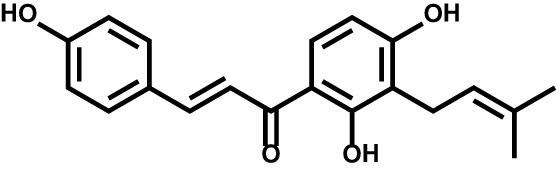	Acute myeloid leukemia	–
Piperine	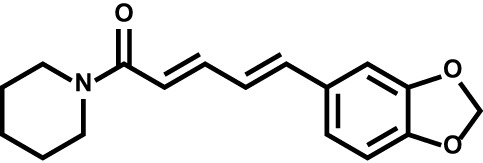	Multiple sclerosis	–

a clinical trial has been terminated due to the lack of sufficient clinical benefit.

## Bioinformatics Analysis of Pyrimidine Metabolism and Signaling in Cancers

**Figure 4:**
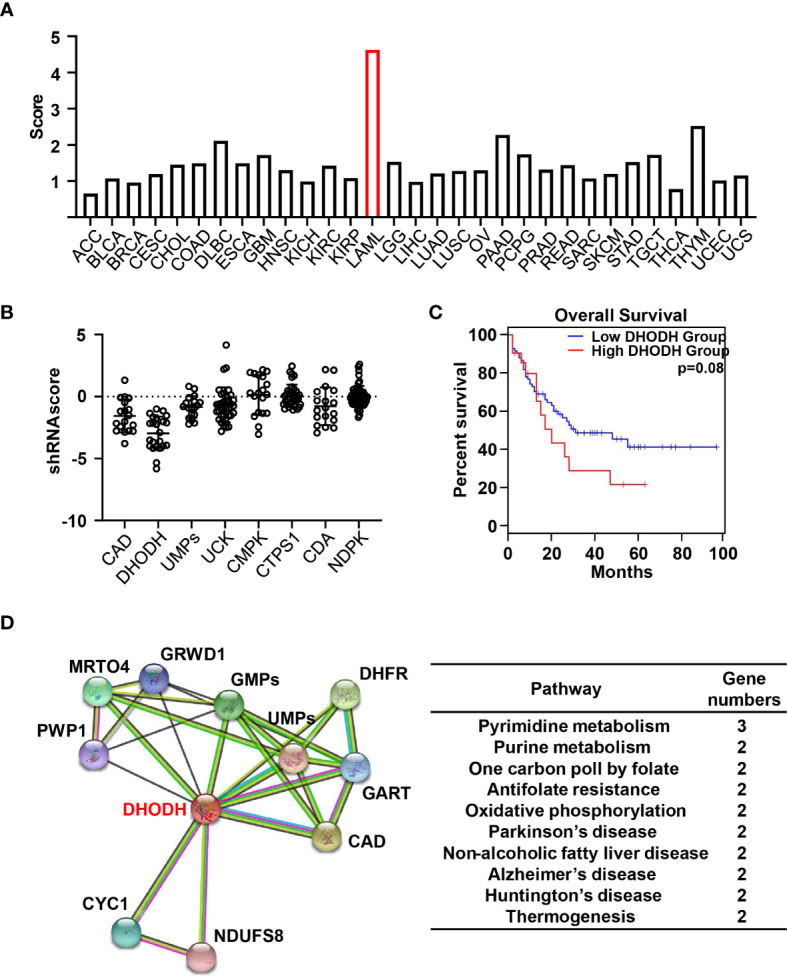
Bioinformatics analysis of pyrimidine metabolism pathway **(A)** Histogram indicate the score of protein abundance of multiple key enzymes involved in pyrimidine metabolism pathway in 31 TCGA tumor tissues compared with corresponding normal tissues in GEPIA. The gene set includes CAD, DHODH, UMPs, UCK1, CMPK, NDPK, CTPS, SLC28A1, SLC28A2, SLC28A3, SLC29A1, SLC29A2, SLC29A3, SLC29A4. **(B)** Dot plots demonstrate the shRNA scores in AML cell lines in CCLE targeting multiple key enzymes involved in pyrimidine metabolism pathway. **(C)** Survival plots suggest the disease-free survival (DFS) for the low and high expression groups of DHODH among AML patients with median cutoff in GEPIA database. **(D)** Predicted the proteins interacted with DHODH according to the STRING V11.0 software and clustered these enzymes through KEGG pathways.

We then turn to investigate the gene expression profiling of pyrimidine metabolizing enzymes in cancer genomics. We first score the pyrimidine metabolism pathway in 31 TCGA cancers compared with corresponding normal tissues in GEPIA (140). As shown in **Figure 4A**, more than 80% cancers demonstrate significantly increased expression of pyrimidine metabolism related genes compared to normal tissues, which indicates the closing correlation of pyrimidine metabolism in tumor progression. Specifically, AML exhibits the highest score.

In order to further examine the importance of pyrimidine metabolism in AML, we then analyzed the cellular responses in the presence of various shRNA in AML cell lines from Cancer Cell Line Encyclopedia (CCLE) (141). As shown in **Figure 4B**, knock down of genes involved in pyrimidine metabolism generally decrease the cell viability, suggesting the indispensable role of pyrimidine pathway in the development of AML. Specifically, the most negative shRNA score occurs in AML is DHODH, suggesting the significant cell proliferation inhibition treated with shRNA. DHODH catalyzes pyrimidine *de novo* pathway simultaneously transferring electrons to mitochondrial respiration chain. Depletion of DHODH significantly disturbs tumor formation with complete functional oxidative phosphorylation. It is reported that DHODH-driven pyrimidine synthesis rather than mitochondrial ATP synthase connects respiration to tumorigenesis (15). Furthermore, DHODH inhibition restructures the ETC upon respirasome assembly and activity owing to the modified phospholipid synthesis dependent on CTP level. Hence, the dual function of DHODH provides the plausible explanation for the prominent effect of shDHODH in AML. Moreover, survival analysis from GEPIA database indicated that higher expression of DHODH was closely associated with shorter disease-free survival (DFS) (**Figure 4C**). These oncology genomics data suggest that DHODH plays a decisive role in the cell survival and proliferation of leukemia.

We then analyzed the potential proteins directly interacting with DHODH using STRING v11.0 and clustered these enzymes through KEGG pathways (142). As shown in **Figure 4D**, in addition to two key enzymes involved in pyrimidine *de novo* pathway, CAD and UMPS, enzymes related with many other cellular metabolism pathways are associated with DHODH, including purine metabolism, one carbon poll by folate, antifolate resistance as well some hall markers in various diseases especially in neurodegenerative diseases. These data suggest that pyrimidine metabolism is paired with other cellular metabolisms and the balance and crosstalk of these metabolism pathway support the cell proliferation and growth. Moreover, patients with high DHODH expression had much worse prognosis than these with lower level, especially in AML. Hence, detecting the expression level of DHODH may provide a promising approach in early diagnosis or predict outcomes in clinical. Taken together, cancer genomic analysis revealed a unique clue for precisely targeting of pyrimidine metabolism pathway in cancer.

## Emerging Therapeutics Targeting Pyrimidine Metabolism in Cancers

### CAD Inhibitors

#### PALA

N-(phosphonacetyl)-l-aspartate (PALA) is a potent inhibitor of *de novo* pyrimidine pathway targeting aspartate transcarbamylase domain of CAD (143). PALA had failed in a phase II trial as single agent owing to the non-ideal efficacy as well as serious side effect due to nonspecifically causing DNA damage in normal cells (144). Additionally, PALA was reported to targeting other metabolic enzymes, such as carbonic anhydrase IV (145). Therefore, more potent, and specific CAD inhibitor is needed to reevaluate the feasibility of targeting CAD.

On the other hand, PALA had showed synergistic anticancer effect in drug combination therapy. For example, Kensler et al. have shown that treatment with drug combination of PALA and acivicin resulted in >80% inhibition of Lewis lung carcinoma tumor growth, and 50% increases in life span (146). Besides, PALA showed synergistical cytotoxic effects with Fluorouracil (5-FU) across most human colon cancer cell lines (147).

### DHODH Inhibitors

#### Leflunomide/Teriflunomide

Leflunomide is an effective DHODH inhibitor that has gained FDA approval for the treatment of rheumatoid arthritis and psoriatic arthritis (148). Leflunomide inhibits DHODH by binding to the ubiquinone binding channel and prevents the production of the orotic acid (149). Teriflunomide (also known as A771726), an active metabolite of leflunomide, was approved by FDA for the treatment of relapsing multiple sclerosis (MS) (150, 151).

In preclinical models, leflunomide and teriflunomide showed anti-proliferative effects against cancer cells from various cancer types through targeting DHODH-driven nucleotide pools accessibility (152, 153). Synergy between leflunomide/teriflunomide and genotoxic chemotherapy agents (doxorubicin, cisplatin, etoposide, and topotecan) was observed by exacerbating DNA damage and overwhelming the DNA damage response (154). In recent years, leflunomide was advanced into clinical trials of solid tumor and hematologic tumor: metastatic TNBC (NCT03709446) and Plasma Cell Myeloma (NCT04370483).

#### Brequinar

Brequinar is a potent and specific inhibitor of DHODH, originally developed by DuPont Pharmaceuticals (DUP 785; NSC 368390) (155) with an IC_50_ of ~20 nM upon DHODH *in vitro* and has a *in vivo* half-life of ~12 hr. Brequinar has been advanced into early clinical trials of patients with advanced solid tumor malignancies, whereas serious toxic effects limit its clinical development (156–159). In 2016, a pioneering study from David B. Sykes group have revealed the excellent therapeutic benefit and safety of brequinar in treating leukemia using an improved dosage regimen in preclinical models. Inspired by these evidences, DHODH targeting by brequinar was recently re-evaluated towards AML in clinical (NCT03760666).

#### Other Synthetic DHODH Inhibitors in Clinical

Recently, Bayer AG have reported a highly potent and selective DHODH inhibitor BAY 2402234, which exhibits strong differentiation therapy potential in preclinical leukemia models. However, the clinical trial has been terminated due to the lack of sufficient clinical benefit (NCT 03404726) (137). ASLAN003, a DHODH inhibitor developed by ASLAN Pharmaceuticals (Singapore, SG), was initial designed for the treatment of autoimmune disease. Subsequent studies have shown that ASLAN003 can also induce leukemia cell differentiation *in vitro* and *in vivo*, providing a rational basis for clinical application of ASLAN003 in leukemia (NCT 03451084) (136). PTC299, an inhibitor of VEGFA mRNA translation, was subsequently identified as a DHODH inhibitor. The unique dual-mechanism of PTC299 may provide a promising therapeutic opportunity for patients with refractory cancers (NCT 03761069) (138).

#### Natural Product-Derived Inhibitors

Natural products represent a critical and valuable source of lead compounds and drugs. Currently, only few natural-occurring compounds have been identified as DHODH inhibitor. We previously reported that isobavachalcone (IBC), derived from Traditional Chinese Medicine *Psoralea corylifolia*, inhibits DHODH with high affinity. IBC dramatically triggers apoptosis as well as overcomes differentiation blockade of multiple AML cell lines. Additionally, the combination of IBC and adriamycin effectively prolong mouse survival in a mouse model of AML (160). On the other hand, using a panel of biochemical assays and structural biology approach, we identified that piperine, a main bioactive constituent of black pepper, as a potent inhibitor of DHODH. We characterized that piperine impairs T cell proliferation as well as reduced inflammation in MOG-induced EAE mouse model with lessened myelin and bold-brain barrier (BBB) destruction by targeting DHODH (161). Collectively, these natural products-derived DHODH inhibitors may represent promising treatment strategy across various diseases.

### UMPs Inhibitors

#### Pyrazofurin

Pyrazofurin was found to inhibit the orotidine 5′-monophosphate decarboxylase activity of UMPS as a nucleoside analogue in the last century with high efficacy especially in acute myelogenous leukemia patients (162). Nevertheless, the resistance to pyrazofurin appeared early in clinical (163–165).

### Other Inhibitors

#### Fluorouracil/Floxuridine/Gemcitabine

Fluorouracil is an analogue of uracil with a fluorine atom at the C-5 position in place of hydrogen, which mimicked uracil incorporating into DNA and RNA, producing intracellularly several active metabolites: fluorodeoxyuridine monophosphate (FdUMP), fluorodeoxyuridine triphosphate (FdUTP) and fluorouridine triphosphate (FUTP) (166). Fluorouracil is reported to bind the deoxyribonucleotide of the FdUMP and the folate cofactor, N5–10-methylenetetrahydrofolate, to thymidylate synthase (TS) to form a covalently bound ternary complex. Up to now, 5-FU has been approved for treating multiple solid tumors including breast, pancreas, stomach, head and neck, and colorectal cancers (167).

Floxuridine is a pyrimidine analogue used as an antineoplastic antimetabolite that is metabolized to fluorouracil, usually as a continuous hepatic arterial infusion to treat hepatic metastases from colon cancer (168). Unlike 5-fluorouracil (5-FU), floxuridine is specifically incorporated into DNA, not into RNA. Inhibition of cell proliferation resulted by floxuridine is 10- to 100-fold higher than that of 5-FU (169, 170).

Gemcitabine is a deoxycytidine analogue, a pyrimidine antimetabolite related to cytarabine. Gemcitabine is converted into phosphate metabolites, which are incorporated in the metabolism of pyrimidine bases and disturb DNA synthesis. Gemcitabine exhibits cell phase specificity, primarily killing cells undergoing DNA synthesis (S-phase) and blocking the progression of cells through the G1/S-phase boundary. In clinical, gemcitabine has been approved to treat various cancers including non-small-cell lung cancer, pancreatic cancer and gallbladder cancer (171–173).

### Drugs Resistance and Potential Combination Therapy in the Future

A wealth of clinical studies has demonstrated the strong tolerance and toxicity of multiple inhibitors of pyrimidine pathway, highlighting the urgent need of improved strategy targeting pyrimidine metabolism. Herein, we mainly summary the emerging role of DHODH inhibition in drugs resistance and combination therapy.

DHODH inhibition can sensitize cancer cells to conventional chemotherapy and overcomes corresponding resistance mechanisms by targeting metabolic dependencies. It is reported that pretreatment with leflunomide induced pyrimidine depletion in TNBC cells and overcame doxorubicin resistance (22) and brequinar was reported to increased cell sensitivity to TRAIL therapy (174). Pyrimidine depletion induced by leflunomide may result in a higher incorporation of chemotherapeutics gemcitabine (175).

KRAS mutant driven cancer cell lines exhibit synthetic lethal vulnerability towards pyrimidine biosynthesis (119). For instance, DHODH inhibitors affect energy metabolism as well as glutamine levels in KRAS mutant cell lines with high sensibility (176). As the potential ability to increase p53 synthesis, DHODH inhibition could combine the inhibitors of p53 degradation and enhance the antitumor effect (177). Therefore, DHODH inhibitors combining traditional chemotherapeutic as well as targeted drugs may increase effectiveness and control unwanted adverse side effects in clinical. Nevertheless, the design of efficient combination projects across multiple cancers types remained to be undeniably challenging.

## Conclusion and Prospects

Metabolic dysregulation has been identified as an emerging hallmark of cancer. A wealth of evidence has demonstrated that metabolic dependencies and phenotypes can evolve in cancer progresses from premalignant lesions to clinically evident cancers to metastasis malignancies. Therefore, understanding sophisticated cancer metabolism and identifying liabilities will expedite the development of new therapeutics to treat human cancer.

Pyrimidine is a basic and indispensable substrate for nucleic acids, phospholipid, glucose metabolism, and protein glycosylation. Recent works in pyrimidine metabolism intended to focus on assessing the interplay of metabolic phenotypes and intrinsic genetic alternation in cancer. It was identified that KRAS mutant, PTEN deficiency as well as p53 deficiency cells exhibits increased pyrimidine *de novo* synthesis flux. This dependence on pyrimidine pathway leads to the synthetic lethal target of pyrimidine synthesis in these cells, suggesting the hardwired metabolic vulnerabilities in different gain-of-function mutant cancers. The crosstalk between pyrimidine pathway and other metabolic signaling generates a greater understanding for metabolic heterogeneity and devises approaches to targeting for clinical therapy. Hence, the discovery of treatments targeting precise pyrimidine metabolism could be done once the underlying metabolic difference is recognized in individual patients.

Considering the crucial role of DHODH in rapidly proliferating cells (like lymphocytes), pharmacological targeting DHODH has revealed ideal effect in inflammatory disease, and autoimmune disease. Importantly, increasing evidence have demonstrated that DHODH closely correlates with various oncogenic signaling pathways in many cancers as discussed above. In clinical, DHODH is actively explored as a target in AML differentiation therapy with several inhibitors advanced in multiple clinical trials. Notably, the development of innovative biomarkers of targeting DHODH in AML is critical for precision or personalized medicine. On the other hand, combination therapy of DHODH inhibitors and conventional chemotherapeutics/targeted agents may offer superior clinical strategies for refractory/resistant AML.

Besides, it is reported that DHODH regulates β-catenin pathway through interacting with NH_2_ terminal of β-catenin directly independent on catalyzed activity (178), suggesting the multi-function properties of DHODH protein. Thus, more diverse, and precise targeting strategies, such as proteolysis-targeting chimera (PROTAC) and molecular glue, are needed to negate the pro-tumor activity of DHODH.

In summary, pyrimidine metabolic remodeling facilitates tumor progression and introduces metabolic vulnerability that can be intervened to treat cancer. It should be noted that cancer metabolism is heterogeneous and flexible, and classical “one-size-fits-all” treatment may fail to achieve satisfactory clinical benefit. With the development of cancer genomics, proteomics, and metabolomics, we prospect that the detailed characterization of the interplay between cellular metabolism and oncogenic signaling will facilitate the development of mechanism-driven precision cancer medicine.

## Author Contributions

WL and JH contributed to conception and design of the review. WW wrote the first draft of the manuscript. JC and HM performed the statistical analysis. All authors contributed to the article and approved the submitted version.

## Funding

This work was funded by the National Natural Science Foundation of China (81973362, 81972828), Shanghai Committee of Science and Technology (18431900500, 19ZR1473500), and Open Research Project of Key Laboratory of High-Incidence-Tumor Prevention & Treatment (Guangxi Medical University), Ministry of Education.

## Conflict of Interest

The authors declare that the research was conducted in the absence of any commercial or financial relationships that could be construed as a potential conflict of interest.
